# The X chromosome and sex-specific effects in infectious disease susceptibility

**DOI:** 10.1186/s40246-018-0185-z

**Published:** 2019-01-08

**Authors:** Haiko Schurz, Muneeb Salie, Gerard Tromp, Eileen G. Hoal, Craig J. Kinnear, Marlo Möller

**Affiliations:** 1 DST-NRF Centre of Excellence for Biomedical Tuberculosis Research, South African Medical Research Council Centre for Tuberculosis Research, Division of Molecular Biology and Human Genetics, Faculty of Medicine and Health Sciences, Stellenbosch University, Cape Town, South Africa; 2 Department of Genetics, St. Jude Children’s Research Hospital, Memphis, TN 38105 USA; 3 South African Tuberculosis Bioinformatics Initiative (SATBBI), Faculty of Medicine and Health Sciences, Stellenbosch University, Cape Town, South Africa

**Keywords:** Tuberculosis, Sex bias, X chromosome, Host genetics, Susceptibility

## Abstract

The X chromosome and X-linked variants have largely been ignored in genome-wide and candidate association studies of infectious diseases due to the complexity of statistical analysis of the X chromosome. This exclusion is significant, since the X chromosome contains a high density of immune-related genes and regulatory elements that are extensively involved in both the innate and adaptive immune responses. Many diseases present with a clear sex bias, and apart from the influence of sex hormones and socioeconomic and behavioural factors, the X chromosome, X-linked genes and X chromosome inactivation mechanisms contribute to this difference. Females are functional mosaics for X-linked genes due to X chromosome inactivation and this, combined with other X chromosome inactivation mechanisms such as genes that escape silencing and skewed inactivation, could contribute to an immunological advantage for females in many infections. In this review, we discuss the involvement of the X chromosome and X inactivation in immunity and address its role in sexual dimorphism of infectious diseases using tuberculosis susceptibility as an example, in which male sex bias is clear, yet not fully explored.

## Introduction

The human sex chromosomes are genomic structures that distinguish males and females on the chromosomal level. The XY sex-determination system is present in humans, and females have two X chromosomes, while males have one Y and one X chromosome [1]. These chromosomes evolved approximately 180 million years ago from ordinary autosomes [2]. Recombination during male meiosis was suppressed, over time, resulting in vast levels of divergence between the human sex chromosomes, with the exception of the pseudoautosomal regions (PAR1 and PAR2) located at the termini of the X and Y chromosomes [3]. Over 800 protein coding and 600 non-coding genes are distributed over the nearly 155 million base pairs of the X chromosome [4]. Until recently the X chromosome has largely been excluded from candidate gene and genome-wide association studies (GWAS) due to the statistical complexity of analysing and comparing the haploid male to diploid female data, but analysis tools have now been developed to incorporate this chromosome.

Gao et al. [5] developed a toolset for X chromosome data analysis and association studies that can be used for quality control and analysis of X chromosome GWAS data. Other software using genotyping data, but not specifically focused on the X chromosome, have also included the option to analyse X-linked genotypes. PLINK version 1.9, a software to conduct association testing using genotyping data, incorporated different models to analyse the X chromosome [6]. Impute2 and shapeit2 are programs designed to impute and phase genotyping data respectively, and until recently, imputation and phasing was not possible for the X chromosome thus excluding this chromosome from downstream analyses [7, 8]. The ability to increase the amount of genotyping data through imputation and including the X chromosome in statistical analysis allows for X-linked meta-analysis and could help elucidate sexual dimorphism. Admixture analysis uses an individual’s genomic data to determine ancestry by comparing allele frequencies to those of reference populations. Until recently, this analysis was inaccurate for haploid genotypes and thus overestimated X-linked ancestral components in males. However, inclusion of haploid-specific ancestry inference in the ADMIXTURE v1.3.0 software now allows for X-linked global ancestry inference [9]. These ancestral components can now be included as covariates in X-linked association testing to improve the quality of the results. The software RFMIX also incorporated the option of assigning local ancestry on the X chromosome [10], allowing the comparison of autosomal and X-linked ancestral distributions, which could indicate sex-biased admixture [11–13].

The development of these tools is especially significant for diseases in which a sex bias is present. Human males are more susceptible to many diseases, including bacterial infections, while females are more likely to develop autoimmunity [14]. This sex bias is not only due to socioeconomic and behavioural factors, such as the underreporting of female cases and/or access to healthcare, but may also in part be due to biological sex differences as determined by the X chromosome and X chromosome inactivation (XCI) [15]. XCI is the process through which one X chromosome is inactivated to balance dosage of gene expression between XX females and XY males. XCI is established early during embryonic development and is maintained almost indefinitely. As males are haploid for the X chromosome, it has been suggested that any damaging genetic variants on the X chromosome will have a more pronounced immunological consequence in males than in females, thereby introducing sex-based differences and influencing the sex bias of a disease. In contrast, females, who are functional mosaics for X-linked genes, may have less-severe consequences, further compounded by the process of skewed XCI and genes escaping silencing [16]. This review will focus on the involvement of the X chromosome and XCI in immunity and will address sexual dimorphism in infectious diseases using tuberculosis (TB) susceptibility as an example, in which sex bias is clear, yet not fully explored.

## X chromosome, the immune system and sex hormones

**Fig. 1:**
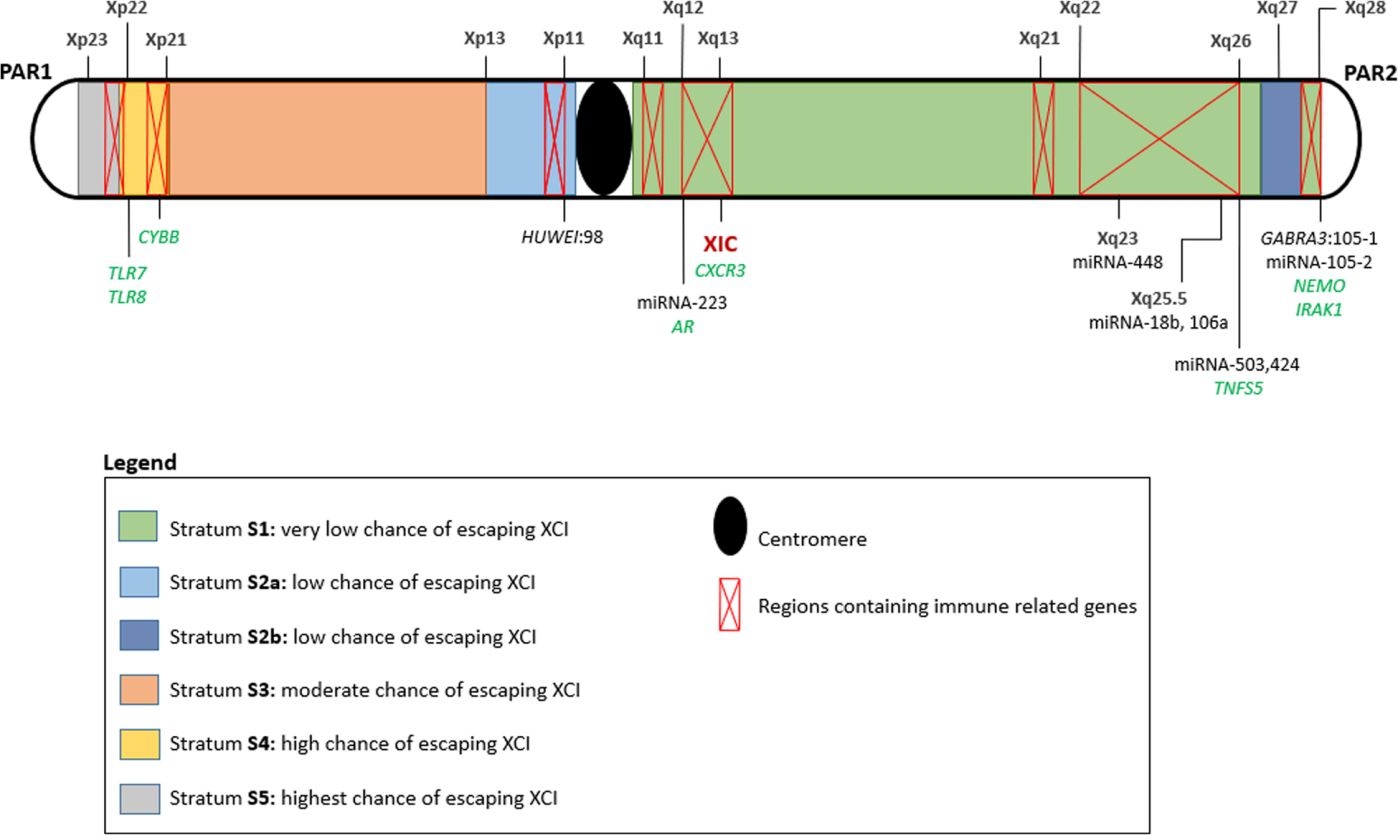
Illustration of the X chromosome indicating the five different strata and chances of genes escaping inactivation within each stratum. Regions lined in red contain the highest densities of immune-associated genes while genes discussed in this review are indicated in green. Genes that contain intragenic miRNA are indicated in black followed by the miRNA number. XIC: X chromosome inactivation centre containing *XIST* and *XACT* genes; PAR: Pseudoautosomal region; *TLR8*: Toll-like receptor 8; *TLR7*: Toll-like receptor 7; *CYBB*: Cytochrome b-245, beta polypeptide; *AR*: Androgen receptor; *CXCR3*: C–X–C motif chemokine receptor 3; *TNFS5*: Encodes CD40 ligand; *NEMO*: NF-kB essential modulator; *IRAK1*: Interleukin-1 receptor associated kinase 1; *HUWE1*: HECT, UBA & WWE domain containing 1; *GABRA3*: Gamma-aminobutyric acid A receptor subunit alpha 3

Many X-linked genes are involved in the innate and adaptive immune system [17]. This includes pattern recognition receptors (PRRs) such as toll-like receptor (*TLR*) *7* and *TLR8* as well as *IRAK1*, a key regulatory molecule in the TLR-dependent signalling pathway [18]. A number of transcriptional and translational control effectors functioning downstream of activated cytokine receptors are also located on the X chromosome [19]. For example, NF-kB essential modulator (NEMO) modulates NF-kB expression, a transcription regulator that is involved in multiple immune pathways [20]. Furthermore, it is not only X-linked genes that could influence the sex bias, but also X-linked control mechanisms like non-coding micro RNA (miRNA). The X chromosome contains approximately 10% of the total genomic miRNA [21], which is involved in the regulation of gene expression by supressing mRNA translation or triggering mRNA degradation. Locations of immune-related genes and key miRNA regions are indicated in Fig. 1.

The androgen receptor, a sex hormone receptor that inhibits antibody production, is also coded on the X chromosome, showing that even the effect of sex hormones can be amplified by the X-linked sex hormone receptor genes [19]. Sex hormones are involved in the immune response, and multiple immune-related cells, including T cells, B cells, natural killer cells, macrophages and dendritic cells, express estrogen receptors (ER-alpha and ER-beta), indicating that immune-related cells are partly controlled by the female sex steroid hormone estrogen [19, 22, 23]. In humans, it is evident that females have increased resistance against microbial infections, which suggests that females have a more vigorous immune defence against most invading pathogens [24–27]. Females also have higher antibody responses and more adverse reactions in response to a number of vaccines [19]. Estrogen acts as an immune activator while testosterone acts as an immune suppressor [19, 28]. Testosterone has been shown to have an inhibitory effect on the immune system through upregulation of anti-inflammatory cytokines (IL-10), while estrogen enhances the immune system by upregulating pro-inflammatory cytokines (TNFα). In line with these hormone functions, it has been observed that for some diseases the male bias becomes apparent only after sexual maturation (ages 15–16 years) and female progression to disease and mortality rates are altered during their reproductive years [29]. However, sex-based differences in immune responses exist between pre-pubertal girls and boys as well as post-menopausal women and elderly men, indicating that sex bias is present without the involvement of hormones [19]. These differences could be attributed to the complexity of studying the impact of hormones on disease susceptibility while using different experimental designs between studies [14]. Sex hormones also vary with age and physiological state of the individual and can regulate transcription of many genes involved in the development and maturation of immune cells. They also influence the regulation and modulation of the immune response and immune signalling pathways [30]. Although both sex-hormones and the X chromosome affect the immune system, the effects of these two factors are likely independent of each other [14].

## X chromosome inactivation

Females carry both a maternal and paternal X chromosome, while males carry only a maternal copy. In order to regulate dosage expression of X-linked genes, one X chromosome is inactivated in females, resulting in them being functional mosaics for X-linked genes [21]. XCI is initiated in early foetal development and either the maternal or paternal X chromosome is randomly silenced in XX cells. This is maintained through epigenetic mechanisms in subsequent cellular divisions to ensure balanced expression X-linked genes in females [31].

XCI developed as a response to gene loss in the Y chromosome during the evolutionary development of the human sex chromosomes [3]. Mammalian sex chromosomes developed from a pair of autosomes approximately 300 million years ago [32]. Several large-scale chromosomal inversions on the Y chromosome led to disruption of homology between the sex chromosomes, suppressing recombination and resulting in Y chromosomal gene loss in the inverted chromosomal region [3]. These inversions on the Y chromosome are referred to as strata as indicated in Fig. 1. Following gene loss on the Y chromosome X-linked gene expression needed to be increased in males to control the dosage of gene expression from the single X chromosome. In females, upregulation of X-linked genes would disrupt dosage compensation as they have two X chromosomes and as a result one X chromosome is inactivated. However, gene expression is upregulated on the active X chromosome in order to regulate dosage [33, 34]. XCI is a vital mechanism in females as many X-linked genes are extremely dosage sensitive and any disruption of the dosage compensation mechanism could have severe developmental and health consequences [33].

Mary Lyon first proposed the XCI hypothesis based on her observations in mice [35], and since then, significant progress has been made in elucidating the XCI mechanism in mice. The XCI mechanism in humans is still unclear and beyond the scope of this review but discussed elsewhere [33, 36–42]. Briefly, human XCI is thought to be controlled by the X inactivation centre (XIC), an X-linked locus located at Xq13 (Fig. 1) and containing multiple protein and RNA coding genes potentially involved in the XCI mechanism [43]. The two main long noncoding RNAs identified thus far are the X inactivation specific transcript (*XIST*), responsible for silencing, and the X active specific transcript (*XACT*) which keeps the X chromosome active [44–47]. The exact mechanisms of how these lncRNAs determine the state of a X chromosome is still unclear, and it has also been proposed that a third regulatory element, potentially coded by a gene on chromosome 19, is also involved in the XCI process [33]. Hypotheses about the lncRNAs as well as an autosomal regulatory element are discussed in detail elsewhere [33, 43, 48–51]. While the exact mechanisms are unclear, the importance of these lncRNAs has been validated as single nucleotide polymorphisms (SNPs) or mutations in the XIC can have severe effects on XCI, by disrupting dosage compensation, which could impact on female development and health [33, 52]. In fact, evidence of the effect of XCI can be seen in tumorigenesis and noncongenital diseases, where loss of XCI control has led to tissue instability and decreased defence against diseases [53–55], including autoimmune diseases [56].

While disruption of XCI could be detrimental to females as it disrupts dosage compensation, the mosaic nature as a result of XCI could give them a distinct advantage over males [14, 37]. Deleterious X-linked mutations have large effects and could lead to death or disease in males due to them being haploid for X-linked genes. In females, however, random inactivation leads to a mosaic makeup where about half of the cell population expresses the mutant allele while the other half expresses the wild type allele. This heterozygous expression means the wild type allele can compensate for the mutant allele and lessen the impact or penetrance of this allele in females compared to males [33]. This mosaic advantage in heterozygous females can be further compounded by non-random or skewed inactivation and genes that escape silencing.

## Escaping X inactivation and skewed or non-random inactivation

While the XCI process in humans is not yet fully understood, studies of human aneuploidy indicate that in a diploid human cell there is always just one active X chromosome in either sex [33, 37]. In Turner syndrome, individuals have only one sex chromosome (one X chromosome, X0) which is kept active, while in males with Klinefelter syndrome (XXY) one X chromosome is silenced [33]. This suggests that the human XCI mechanisms protect one X chromosome while inactivating all others.

However, some X-linked genes have Y homologues (most of them situated on the distal end of Xp and PAR regions) and thus two copies are present in males and females. To maintain dosage balance between the sexes, these XY genes escape silencing. Most genes that escape silencing are located in the Xp region and are often depleted in repressive marks associated with XCI and enriched for markers associated with active gene transcription [57]. These regions that escape inactivation carry features associated with active chromatin [58]. This suggests that genes that escape silencing are subjected to a regional bias, which correlates with the theory that distal genes in younger strata (regions on the X chromosome that differentiated from the Y chromosome last and contain more XY genes than older strata) have a higher chance of escaping inactivation.

More recent evidence extrapolated on the idea of regional bias in escape from inactivation and showed that the chance of genes escaping silencing is also dependent on a gene to gene-specific bias [32]. This is supported by the fact that approximately 15–20% of X-linked genes outside of PAR also escape silencing even though they are subject to less regional bias. Naqvi et al. [32] classified X-linked genes into three classes, namely X-linked genes with a surviving homologue (class 1) and X-linked genes without a surviving homologue that are either subject to XCI (class 2) or escape silencing (class 3) [32]. These three classes of X-linked genes differ based on dosage sensitivities. Class 1 genes were most dosage sensitive and expression required strict regulation, while class 2 genes had intermediate dosage sensitivity while class 3 genes that escaped silencing had the lowest dosage sensitivity [32]. This suggests that genes that escape silencing are subjected to regional bias and the chance of escape depends on the sensitivity of that gene to changes in dosage. While defects in the XCI mechanism could disrupt the XCI pattern of dosage-sensitive genes and be detrimental to the health and development of females, genes that are less sensitive to dosage could escape resulting in altered gene expression between the sexes and potentially contribute towards a sex-specific phenotype, which could contribute to sex biases in disease susceptibility [14, 17, 59].

Random inactivation ideally leads to a balanced mosaic of X-linked genes in females. However, this balance can be disrupted, especially in heterozygous females carrying deleterious mutations on one or both X chromosomes, or if the XCI mechanism is defective, leading to a skewed inactivation pattern. Skewed inactivation is the process by which one X chromosome is preferentially silenced in over 75% of cells. If a cell has a deleterious mutation on the active X chromosome, it could alter the viability of the cell and even lead to cell death, suggesting that these mutations could lead to positive or negative selection of a specific active X chromosome [60, 61]. The extent of this selection pressure is correlated with three factors. Firstly, the viability of the cell which will be determined by the active X chromosome. If cells with an active X chromosome with a detrimental gene die, then only cells with the viable gene will propagate. This depends on the type of mutation (synonymous or non-synonymous) and its effect on gene function. Second, the gene function can influence the skewing if it is tissue-specific while a constitutively expressed gene could affect the skewing on a global scale. Finally, genes escaping inactivation can also influence selection as they will influence the penetrance of the mutated gene [62]. While cell viability combined with XCI can skew inactivation patterns, other aspects can also lead to non-random inactivation. Defects in the XCI mechanism can also lead to skewed inactivation and SNPs in the *XIST* gene correlates with skewing. Plenge et al. [52] showed that skewed inactivation profiles in multiple females occurred due to a C to G transversion in the promoter region of the *XIST* gene [52]. However, some females with this transversion still had nearly random inactivation suggesting that the transversion alone is not enough to skew inactivation and some other defect compounding the effects is likely present as well.

Other factors that can result in skewed inactivation are reduced number of embryonic cells at the onset of XCI and age. The lower the number of cells at the onset of XCI, the higher the chance of observing non-random inactivation and any bottleneck during development that limits the number of cells can lead to skewed inactivation [62]. Age has also been correlated with degree of skewing which seems to increase in older women [63–66]. The exact reason why skewing increases with age is unclear, but it could be as a result of stochastic loss and genetic selection of subtle SNPs, gradually increasing their penetrance over time due to increased skewing in the XCI pattern [63, 64, 67, 68]. The causes of skewed XCI discussed here suggest that this process is genetically determined [60] and can give females an advantage by protecting them from deleterious mutations and their effects. However, skewed inactivation patterns have also been observed in numerous tumours and cancer types [57, 69]. This suggests that the combined impact of XCI, genes that escape silencing and skewing can lead to sex-specific phenotypes and potentially affect disease and developmental bias between the sexes.

## X chromosome and infectious disease susceptibility

It is well documented that females have a stronger innate and humoral immune response than males and are thus less susceptible to many bacterial, fungal, parasitic and viral infections, while being more prone to developing an autoimmune disease or malignancies (Table 1, [25]). However, as not every microorganism elicits a sex-differentiated response, it has been proposed that the invading organisms and how they interact with the host are important contributing factors to whether or not the host immune response will differ between the sexes [70].

**Table 1:** Sex bias of selected bacterial, fungal, parasitic and viral infections

Infection	Organism	Disease	Bias	Reference
Bacterial	*Treponema pallidum*	Syphilis	Male	[103–105]
	*Borrelia burgdorferi*	Lyme disease	Male (age)	[71, 72]
	*Vibrio vulnificus*	Infection	Male	[106]
	*Staphylococcus aureus*	Infection	Male	[107, 108]
	*Pseudomonas aeruginosa*	Infection	Male	[107, 108]
	*Escherichia coli*	Bacteraemias	Female	[107, 108]
Fungal	*Cryptococcus neoformans*	Fungal meningitis	Male	[109–111]
	*Candida albicans*	Onychomycosis	Female	[112–117]
	*Paracoccidioidal brasiliensis*	Infect mucosal membranes	Male	[118]
Parasitic	*Schistosoma*	Schistosomiasis	Male	[119–121]
	*Leishmania*	Leishmaniasis	Male	[119–121]
	*Taenia*	Tapeworm	Female	[119–121]
Viral	*Influenza A*	Influenza	Male	[122–125]
	*Hepatitis C*	Hepatitis	Male	[73, 74]

Many infections exhibit sex-biased incidence rates and many of them present with a male bias (Table 1). While age and sex hormones contribute, as in the case of Lyme disease and hepatitis, these factors do not fully account for this [71–74]. This suggests that the X chromosome and XCI may contribute to this bias. Supporting evidence from this can be taken from the mouse four core genotype (FCG) model. In this model, the sex chromosome complement of the mice (XX or XY) does not relate to the gonadal sex, allowing for both XX males and females as well as XY males and females [75]. This allows the study of the phenotypic effect based on sex complement, with and without the influence of sex hormones. Studies using the FCG model have identified differences in behaviour, gene expression and disease susceptibility that were solely due to sex chromosome complement and independent of sex hormones [75].

While the FCG is only a model, it can still provide useful information and shows that sex chromosome complement, X-linked genes and XCI can severely impact sex differences in phenotype. Recent studies in female T and B cells could explain the enhanced female immune response to infection. XCI in female lymphocytes is predisposed to become partially reactivated, allowing genes to escape silencing leading to overexpression of immune-related genes [49, 76]. Female T cells had biallelic expression of *CD40LG*, *CXCR3* and *TLR7*. The same was observed for B cells where biallelic expression and increased transcription of X-linked immune-related genes was observed [76]. Furthermore, in both T and B cells, the *XIST* RNA pattern was dispersed and the inactivated X chromosome lacked typical heterochromatic modifications usually associated with the inactive X chromosome [76].

These studies in female lymphocytes provide mechanistic evidence for enhanced female immunity to infectious diseases and the involvement of X-linked genes and XCI. The enhanced immune response and increased expression of immune-related genes could also explain why females are more prone to developing autoimmune disorders [14, 25, 76].

## X chromosome and tuberculosis

TB, caused by the bacterium *Mycobacterium tuberculosis*, is the leading cause of death due to a single infectious agent worldwide. Approximately one quarter of the world’s population is infected with the bacterium, but only 5–15% will develop active TB [77]. The severity of this pandemic is exacerbated by the emergence of multidrug-resistant and extensively drug-resistant (MDR and XDR) *M. tuberculosis* strains. Although vital to the affected individual, it is clear that antimycobacterial treatment alone will not eradicate this disease. Host-directed therapy is emerging as a complementary approach to reduce the global TB burden, but will require an improved understanding of the host immune response and the genetic mechanisms that underlie it [78]. To date, variants of genes involved in both the innate and adaptive immune responses have been associated with TB (reviewed by [79]). However, these investigations have been largely aimed at the autosome, while excluding the X chromosome. Given the high density of immune-related genes on the X chromosome [19] and the fact that TB presents with a clear sex bias across populations, this is a serious oversight [80].

In most countries, the TB notification rate is twice as high in HIV-negative males than in HIV-negative females [80]. This ratio ranged from 1.56 to 2.73 and while it differs between countries, it was clear that more men than women are affected regardless of ethnicity or geographical location. Epidemiological data has shown that males and females differ in infection prevalence, varying rates of progression, differences in incidence of clinical disease and mortality rates due to TB [81]. The cause of this male sex bias is not fully understood, but may include socioeconomic and behavioural factors, such as the underreporting of female cases and/or access to healthcare [23, 82–84]. However, these differences in case reporting may influence the bias but cannot explain the consistent global trend for male bias in TB [22]. In a large meta-analysis including 29 surveys from 14 countries, a strong male bias was found in both TB notifications and prevalence and it was concluded that access to healthcare is not a confounding factor. This was replicated by Salim et al. [82] who conducted a survey of 223,936 individuals in Bangladesh and identified 7001 TB suspects at a female to male ratio of 0.52:1. Sputum was obtained from these individuals and 64 positive TB cases were identified at a female to male ratio of 0.33:1. These observed ratios did not differ much and were in fact lower than the female to male ratio observed through diagnosis in clinics which stood at 0.42:1. The authors concluded that reduced access of women to health care facilities does not significantly influence the bias seen [82]. In a study conducted in Syria, men and women did not have different knowledge or attitudes towards TB, but women reported more barriers to seeking health care. They were more likely to comply with treatment and had higher treatment success rates compared to men which could influence the bias when it comes to TB mortality [85]. Furthermore, men seem to engage in more “high risk” TB activities, including travelling, smoking, going to bars and hazardous careers (e.g. mining) [22]. In high-burden countries, more men than women engage in smoking and it has been suggested that smoking may explain up to one third of the gender bias observed in TB [86]. Alcohol consumption could have a similar effect. However, other risk factors, specifically HIV infection and proximity to household contacts, appear to have a female bias, which suggests that although behaviour may influence the bias it is not sufficient to fully explain the existing sex bias in TB [22]. Another contributing factor may be the influence of sex hormones on the immune system (discussed in section “X chromosome, the immune system and sex hormones”).

Females have been shown to have a more robust immune system (as described in section “X chromosome and infectious disease susceptibility”), and this is in part mediated by sex hormones that control development and maturation of immune cells (T cells, macrophages, neutrophils) involved in combating TB. Type 1 T helper cells (Th1) are affected differently by male and female sex hormones. Testosterone upregulates IL-10 while downregulating IFN-γ [83] and estrogen increases IFN-γ, TNFα and IL-12 production while supressing production of IL-10 [84]. Macrophages, which play a central role in controlling TB through active killing of mycobacteria, are also influenced by sex hormones. The female hormone estradiol has been shown to enhance macrophage activation [29], while testosterone downregulates macrophage activation by decreasing expression of TLR4, a vital receptor for detecting *M. tuberculosis* and initiating the innate immune response [24]. Neutrophils have recently garnered interest with regard to their role in protection against TB and have been proposed to be the predominantly infected phagocytic cell type in pulmonary tuberculosis (pTB) [87]. Neutrophil recruitment to areas of infection needs to be balanced as under- and over-recruitment of neutrophils can have a detrimental effect on tissue pathology [88]. In response to trauma, testosterone decreases neutrophil activation while estrogen increases it, but the effect of this on TB is unknown and requires further investigation [89]. As neutrophil recruitment needs to be balanced to avoid under- or over-recruitment to sites of infection, it stands to reason that the regulation of this recruiting mechanism is of vital importance. In fact, miRNA-223 (Xq12, Fig. 1), previously identified to be involved in the immune response by Pinheiro et al. [90], can limit recruitment of neutrophils by downregulating chemokine (C–X–C motif) ligand 2 (CXCL2) and chemokine (C–C motif) ligand 3 (CCL3). Mice with a miRNA-223 knockout were more susceptible to *M. tuberculosis*, due to excessive neutrophil accumulation in the lungs which subsequently led to tissue damage [91]. Given that miRNA-233 is X-linked, is subject to the effects of skewed inactivation or may escape silencing, it could be differentially expressed between males and females. Upregulation due to escape from silencing or preferential expression of one gene copy due to skewed inactivation could downregulate recruitment and thus the pathological accumulation of neutrophils leading to a sex bias in TB susceptibility. Clearly, these factors do not fully explain the male bias associated with TB disease development, suggesting that the host genotype, specifically the X chromosome, may also contribute.

The third possible reason for the sex bias in TB susceptibility is linked to the X chromosome where skewed inactivation or genes escaping silencing could give females an enhanced immune response against *M. tuberculosis.* Some of the earliest evidence of this X-linked genetic contribution to sex bias in TB susceptibility came from the “Lübeck Disaster” in 1929. Bacillus Calmette–Guérin (BCG) vaccine accidentally contaminated with *M. tuberculosis* was administered to 251 neonates*.* One hundred seventy-three of these children developed signs of active TB but recovered, while 72 died, and during follow-up, male children were more likely to have poor outcomes than females [92]. Evidence from studies of Mendelian susceptibility to mycobacterial disease (MSMD) also supports the influence of the X chromosome to disease susceptibility. MSMD is a rare congenital syndrome that results in the predisposition to diseases caused by non-virulent mycobacteria, BCG vaccines and environmental mycobacteria known not to be disease causing in humans [93]. MSMD is classified into two types, where autosomal MSMD is linked to defects in five autosomal genes (*IFNGR1*, *IFNGR2*, *STAT1*, *IL12RB1* and *IL12B*) involved in the interleukin 12/23 dependant interferon γ (IFN-γ)-mediated immune response [94]. On the other hand, X-linked recessive (XR)-MSMD is less well understood [20]. Several genetic defects have been proposed to cause XR-MSMD, and based on the genes involved, XR-MSMD can be further subdivided into two types, XR-MSMD type 1 and XR-MSMD type 2. Type 1 XR-MSMD is caused by mutations in the leucine zipper domain of the NF-kB essential modulator (*NEMO*) gene, which selectively impairs the CD40 and NF-kB/c-Rel-mediated induction of IL-12 production by monocytes and monocyte-derived dendritic cells [93]. Predisposition of type 2 XR-MSMD is increased by mutations in two regions on the X chromosome, Xp11.4-Xp21.2 (129 known genes) and Xq25-Xq26.3 (70 known genes). These regions may cause XR-MSMD independent of *NEMO*, and Bustamante et al. proposed that variants in the cytochrome b-245 beta polypeptide (*CYBB)* gene could predispose to XR-MSMD-2 due to their selective effect on macrophages. *CYBB* encodes the gp91 protein, which is an essential component of the NADPH oxidase complex and severely affects respiratory burst in macrophages, thereby impeding their function and predisposing to XR-MSMD-2. *NEMO* and *CYBB* are both X-linked genes that affect immune-related cells and as such can alter susceptibility to TB. XR-MSMD, like TB, shows a sex bias and affects more males than females which can be attributed to females carrying two X chromosomes. If one of the X chromosomes carries a defective *NEMO* or *CYBB* gene, random XCI can result in the functional gene product still being expressed and reducing the risk of disease. Skewed inactivation or escape from silencing could further increase the observed sex bias as *NEMO* and *CYBB* have a low (stratum S1) and high (stratum S4) chance of escaping inactivation (Fig. 1). However, TB in immunocompetent individuals is a multigenic disease linked to variants in multiple genes that have a cumulative effect on disease susceptibility and is even further complicated by gene-gene interactions.

The first genome-wide linkage analysis of TB susceptibility identified the chromosome Xq26 region as containing susceptibility genes, but did not specifically investigate sex bias [95]. Although no specific genes could be identified, the CD40 ligand encoded by the *TNFSF5* gene at Xq26.3 showed promise (Fig. 1), but requires further investigation [95]. A study by Campbell et al. [96] on 121 TB cases and their parents identified a *TNFSF5* (a CD40 ligand) variant (− 726) to be associated with TB susceptibility in males. However, they failed to replicate this association in a West African cohort of 1200 individuals.

More recently, sex-specific associations with genetic variants in the X-linked toll-like receptor (*TLR*) 8 gene (Table 2), which encodes a pattern recognition receptor, were identified [97–102]. Davila et al. [98] identified four variants in *TLR8* (rs3764879, rs3788935, rs3761624 and rs3764880) that were significantly associated with TB susceptibility in Indonesian males, but not females. These findings were validated in a male only cohort from Russia and all four variants were again significantly associated with TB susceptibility in males. A second study conducted in a paediatric Turkish cohort showed a significant association between rs3764880 and TB susceptibility in males but not females and rs3764879 showed no significant association in this cohort [99]. Hashemi-Shahri et al. [100] also investigated the influence of rs3764880 on TB susceptibility in a cohort from Iran but found no association in either males or females. Significant associations were found for both males and females in a Pakistani cohort for rs3764880, but males were more strongly associated (*p* = 0.0013 for females and *p* < 0.0001 for males) [101]. Salie et al. [97] was the first to identify an association between rs3761624 and TB disease in females only (*p* < 0.001 for females and *p* = 0.164 for males). Two SNPs, namely rs3764879 and rs3764880, were also investigated in this South African Coloured (SAC) population and were significantly associated in both males and females, but with opposite effects. Finally, Lai et al. [102] showed that rs3764879 was significantly associated with TB in males but not females. The conflicting results of these studies investigating *TLR8* may be explained by cohort size, ethnicity, *M. tuberculosis* strain and environmental factors.

**Table 2:** TLR8 association studies from different populations

Study	Cohort	Case	Control	SNP	Allele	Gender	OR*	95% CI*	*P* value
Davila et al. [98]	Indonesia	77	49	rs3764879	C	Male	1.9	1.2–2.7	0.012
		76	74	rs3764879	C	Female	1.1	0.8–1.7	0.44
		76	51	rs3761624	A	Male	1.8	1.2–2.8	0.007
		76	74	rs3761624	A	Female	1.1	0.8–1.7	0.44
		76	50	rs3788935	A	Male	1.8	1.2–2.7	0.017
		76	74	rs3788935	A	Female	1.1	0.8–1.7	0.44
		76	51	rs3764880	A	Male	1.8	1.2–2.9	0.007
		76	74	rs3764880	A	Female	1.1	0.8–1.7	0.44
	Russia	1067	994	rs3764879	C	Male	1.2	1.02–1.48	0.03
		1069	997	rs3788935	A	Male	1.2	1.02–1.48	0.03
		1070	1000	rs3761624	A	Male	1.2	1.01–1.46	0.04
		1069	997	rs3764880	A	Male	1.2	1.02–1.48	0.03
Dalgic et al. [99]	Turkish children	72	62	rs3764880	A	Male	0.43	0.16–0.72	0.007
		156	124	rs3764880	A	Female	NS	NS	NS
		72	62	rs3764879	C	Male	NS	NS	NS
		156	124	rs3764879	C	Female	NS	NS	NS
Hashemi-Shahri et al. [100]	Iran	77	62	rs3764880	G	Male	1.15	0.84–1.59	0.80
		196	166	rs3764880	G	Female	1.15	0.75–1.75	0.51
Bukhari et al. [101]	Pakistan	45	22	rs3764880	A	Male	/	/	< 0.0001
		58	65	rs3764880	A	Female	0.363	0.199–0.660	0.0013
Salie et al. [97]	SAC	204	99	rs3761624	A	Male	/	/	0.164
		217	336	rs3761624	A	Female	1.54	1.19–1.99	< 0.001
		205	99	rs3764879	C	Male	0.72	0.55–0.93	0.013
		220	334	rs3764879	C	Female	1.41	1.08–1.83	0.011
		1887	81	rs3764880	A	Male	0.75	0.57–0.98	0.036
		199	306	rs3764880	A	Female	1.42	1.09–1.87	0.011
Lai et al. [102]	Chinese	96	146	rs3764879	C	Male	4.04	1.82–8.99	< 0.001
		40	97	rs3764879	C	Female	5.05	0.44–57.38	0.191

*OR* odds ratio; 95% *CI* 95% confidence interval

It is clear that the X chromosome and XCI (section “X chromosome and infectious disease susceptibility”) is significantly involved in TB susceptibility and the male sex bias and future studies will need to focus on elucidating these effects. Fully understanding the sex-biased nature of TB will allow for medication tailored to a specific sex, which could improve treatment outcome, decrease the global TB burden and stem the tide of emerging drug-resistant *M. tuberculosis* strains.

## Discussion and concluding remarks

It is clear that sex-specific effects contribute to infectious disease susceptibility and females have a major immunological advantage over males. Understanding the origin of sex bias could guide treatment by allowing sex-specific diagnostic and treatment regimes, thereby decreasing time to initiation of treatment as well as increasing treatment success of diseases with sex differences. The X chromosome may contribute to the missing heritability or contain biomarkers that could be used as diagnostic tools. As analytical tools are now available to fully include the X chromosome in genetic analyses, it is clear that the X chromosome should not be ignored. Importantly, due to the haploid nature of males, the power to detect a significant association will be halved when compared to a female cohort of similar size and this could have an effect on the results of sex-stratified analysis. Thus, care must be taken when analysing results, and a non-significant association in one sex does not imply that that specific sex is not affected by the variant, but could simply be as a result of insufficient power to detect a sex-specific association.

While socioeconomic and behavioural factors as well as sex hormones do influence sex bias, these factors do not fully account for it, which leads to the conclusion that the X chromosome itself is likely to greatly influence the immune response and sex bias in disease susceptibility. The X chromosome contains multiple immune-related genes and immune regulatory elements as well as the XIC that regulates X chromosome inactivation. It is therefore clear that the X chromosome is involved in the immune response and genes that escape inactivation or are preferentially inactivated could influence the dosage of X-linked gene expression between the sexes and as such could further influence the sex bias in disease. It is thus of vital importance that the XCI mechanisms be further investigated to understand all the regulatory elements involved and the contribution to sex bias. Furthermore, the role of the X chromosome in the innate and adaptive immune response should be extensively investigated to determine how it contributes and differs between the sexes. Elucidating the function of the X chromosome and including it in biological studies and analyses could improve the understanding of complex diseases such as TB.

## Abbreviations

BCG: Bacillus Calmette–Guérin
CI: Confidence interval
CYBB: Cytochrome b-245 beta polypeptide
ER: Estrogen receptors
GABRA3: Gamma-aminobutyric acid A receptor subunit alpha 3
GWAS: Genome-wide association studies
HIV: Human immunodeficiency virus
*HUWE1*: HECT, UBA & WWE domain containing 1
IFN-γ: Interferon gamma
IL: Interleukin
L1: Long interspersed nuclear elements
lncRNA: Long non-coding RNA
MDR: Multidrug-resistant
miRNA: Micro RNA
mRNA: Messenger RNA
MSMD: Mendelian susceptibility to mycobacterial diseases
NADPH: Nicotinamide adenine dinucleotide phosphate hydrogen
NEMO: NF-kB essential modulator
NF-kB: Nuclear factor kappa-light-chain-enhancer of activated B cells
OR: Odds ratio
PAR: Pseudoautosomal region
PRR: Pattern recognition receptors
pTB: Pulmonary tuberculosis
RNA: Ribonucleic acid
SNP: Single nucleotide polymorphism
TB: Tuberculosis
Th1: T helper cells
TLR: Toll-like receptor
TNFS5: Encodes CD40 ligand DNA (deoxyribonucleic acid)
TNFα: Tumour necrosis factor alpha
XCI: X chromosome inactivation
XDR: Extensively drug-resistant
XIC: X inactivation centre
XR: X-linked recessive
